# Changes in precipitation amounts and extremes across Xinjiang (northwest China) and their connection to climate indices

**DOI:** 10.7717/peerj.10792

**Published:** 2021-01-25

**Authors:** Wenfeng Hu, Junqiang Yao, Qing He, Jing Chen

**Affiliations:** 1 Fuyang Normal University, History, Culture and Tourism School, Fuyang, China; 2 Institute of Desert Meteorology, China Meteorological Administration, Urumqi, China

**Keywords:** Extreme precipitation indices, Continuous wavelet transform, Probability distribution functions, Climate indices, Xinjiang

## Abstract

Xinjiang is a major part of China’s arid region and its water resource is extremely scarcity. The change in precipitation amounts and extremes is of significant importance for the reliable management of regional water resources in this region. Thus, this study explored the spatiotemporal changes in extreme precipitation using the Mann–Kendall (M–K) trend analysis, mutation test, and probability distribution functions, based on the observed daily precipitation data from 89 weather stations in Xinjiang, China during 1961–2018. We also examined the correlations between extreme precipitation and climate indices using the cross-wavelet analysis. The results indicated that the climate in Xinjiang is becoming wetter and the intensity and frequency of extreme precipitation has begun to strengthen, with these trends being more obvious after the 1990s. Extreme precipitation trends displayed spatial heterogeneity in Xinjiang. Extreme precipitation was mainly concentrated in mountainous areas, northern Xinjiang, and western Xinjiang. The significant increasing trend of extreme precipitation was also concentrated in the Tianshan Mountains and in northern Xinjiang. In addition, the climate indices, North Atlantic Oscillation, Atlantic Multidecadal Oscillation, Multivariate ENSO Index and Indian Ocean Dipole Index had obvious relationships with extreme precipitation in Xinjiang. The relationships between the extreme precipitation and climate indices were not clearly positive or negative, with many correlations advanced or delayed in phase. At the same time, extreme precipitation displayed periodic changes, with a frequency of approximately 1–3 or 4–7 years. These periodic changes were more obvious after the 1990s; however, the exact mechanisms involved in this require further study.

## Introduction

Global warming is an indisputable objective fact (IPCC, 2013). Within the context of global warming, extreme climate events (e.g., temperature and precipitation extremes) vary in their frequency or intensity and can affect the natural environment and human lifestyles, even having political consequences. Therefore, numerous studies have been conducted to investigate weather extremes, with many of these focusing on the study of extreme precipitation. Precipitation extremes increase with warming because of increases in the saturation vapor pressure of water (Allen & Ingram, 2002; O’Gorman & Schneider, 2009; Trenberth et al., 2003; Van den Besselaar, Klein Tank & Buishand, 2013; Kao & Ganguly, 2011; Xu et al., 2011; Zhang et al., 2019). Researchers have reported that extreme precipitation shows a clear increasing trend in North America (Griffiths & Bradley, 2007) and Europe (Moberg & Jones, 2005), while precipitation in arid regions has decreased and there are significant regional differences (Hulme, 1996), at the same time, extreme precipitation events have increased (Donat et al., 2017). The IPCC (2013) also reported that extreme precipitation events have become more intense and more frequent in most mid-latitude areas in the context of global warming.

The vast majority of China’s territory lies in the mid-latitudes of the Northern Hemisphere and is faced with the threat of extreme weather events. Many studies in China have been conducted to determine changes in extreme precipitation. They have reported that precipitation has significantly increased in Northwest China, with an increasing trend in extreme annual precipitation in areas where the daily precipitation exceeds 10 mm (Sun, Liu & Gao, 1998; Zhai et al., 1999). There is real evidence of an increased intensity and frequency of various types of extreme precipitation in recent decades (Zhang et al., 2011a). Due to the arid and semi-arid regions of China being particularly sensitive areas in terms of their response to climate change (Ding et al., 2007; Shi et al., 2007), Northwest China has become an important area of concern for researchers. A dramatic change has been identified, in which the warm-dry regional climate shifted toward a warm-wet climate during the 1980s (Shi, Shen & Hu, 2002; Chen et al., 2006). Wang et al. (2013) reported that cold index values have continually declined, while warm index values have risen, the daily differences in temperature have continually decreased and most precipitation indices have displayed increasing trends.

Xinjiang, in the hinterland of the Eurasian continent, is located in inland China, an arid region of the Northern Hemisphere, which is very sensitive to global warming (Yao et al., 2018). Studies have shown that precipitation has continually decreased in southern Xinjiang and continually increased in northern Xinjiang (Wang et al., 2013). The severity of drought has reduced and drought duration is also shortening in north Xinjiang. However, drought has intensified in the central part of east Xinjiang and the southern part of south Xinjiang (Zhang et al., 2012a). Some studies have reported that the climate has shown a tendency to be wetter after 1997 in Xinjiang (Yao et al., 2018). Some scholars found that there were existed apparent regional differences in precipitation extremes across Xinjiang (Zhang et al., 2012b; Deng et al., 2014), wetter trends and precipitation extremes increased in the Tianshan Mountains and most of Xinjiang (Zhang et al., 2019). Another scholar pointed out the increasing trends in northern Xinjiang and exhibit decreasing trends in southern Xinjiang (Wang et al., 2013). However, studies have also shown that the increasing trends of the precipitation extremes were observed mainly in the northern Xinjiang and the north of the southern Xinjiang, and most extreme precipitation indices show a potential regime shift starting from the middle of 1980s (Jiang et al., 2013), Chen et al. (2014) also pointed out that abrupt change in precipitation extremes in Northwest China occurred in around 1986, and the Index B of the Tibetan Plateau (TPI_B) was probably an important factor in the abrupt change in precipitation extremes in the Xinjiang (Northwest China). Although some researchers have studied the spatio-temporal features of extreme precipitation in Xinjiang (Jiang et al., 2013; Deng et al., 2014), our understanding of the changes of extreme precipitation is insufficient. The key purpose of our study is to determine the spatial distribution and temporal trends of precipitation extremes in Xinjiang from 1961–2018, and to conduct an in-depth analysis of the periodicity of extreme precipitation and the relationships between changes in extreme precipitation events and climate indexes.

## Materials and Methods

### Data and Selected indices

**Figure 1:**
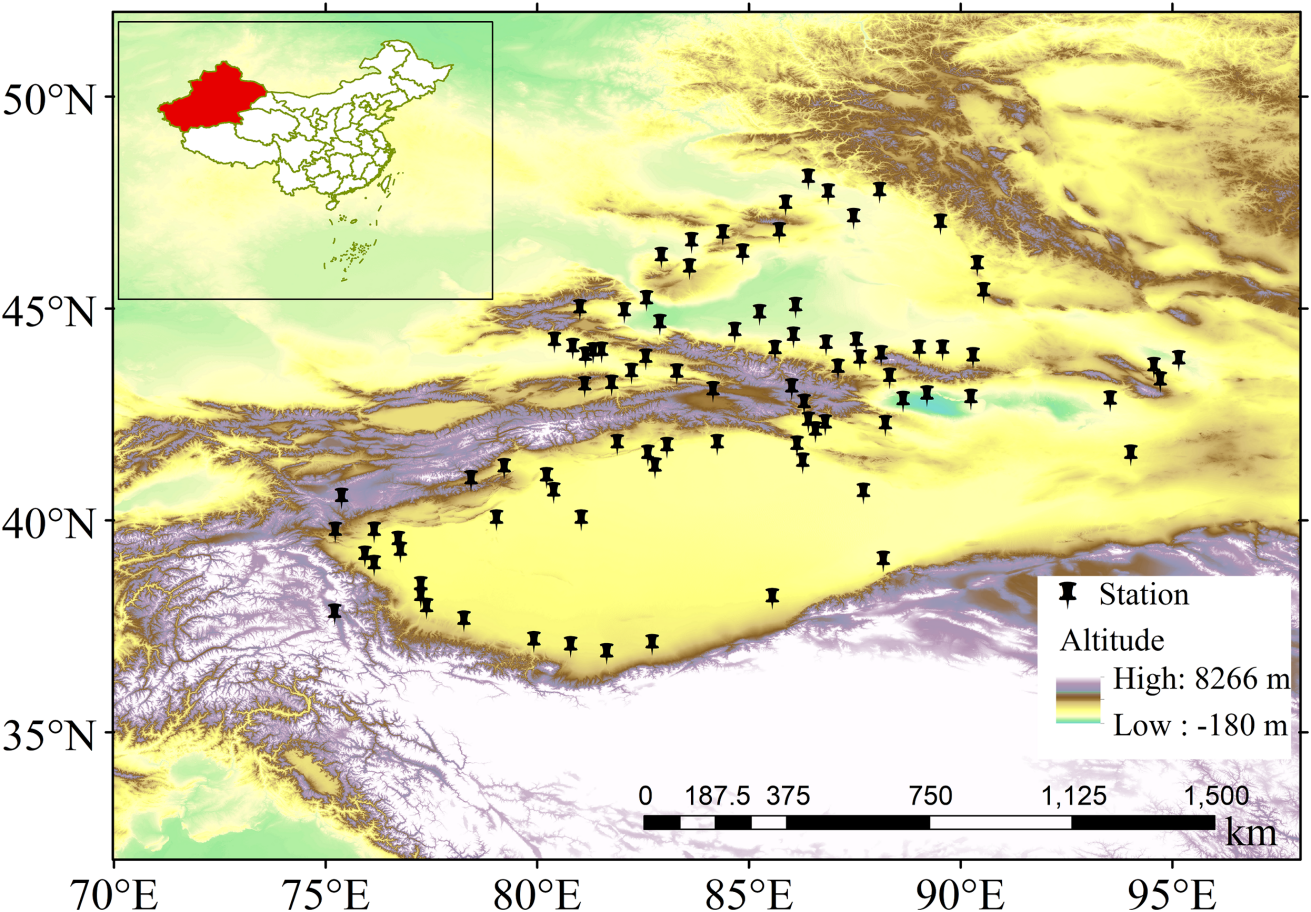
Map of the study area and stations. The black nails represent the weather stations.

**T**he meteorological data from 107 weather stations in Xinjiang were provided by the China Meteorological Data Network (http://data.cma.cn/data/cdcdetail/dataCode/SURF_CLI_CHN_MUL_DAY.html). After accounting for missing data and comparing the length of the recorded period, we then selected 89 stations for the period from 1961 to 2018. Missing values account for >1.5% of in the daily precipitation series at 4 stations and <1.5% at other stations. The missing data were completed using conventional statistical methods including: (1) if only one day has missing data, the missing data was replaced by the average value of its two nearest stations; (2) if consecutive two or more days have missing data, the missing data would be processed by simple linear correlation between its nearest stations. These missing data and interpolation have no effect on the results of our study. The selected stations were relatively evenly distributed, which enabled the regional precipitation changes in Xinjiang to be reflected (as shown in Fig. 1). Data quality control and calculation of the extreme precipitation index from the original datasets were undertaken using the R ClimDex software (http://etccdi.pacificclimate.org/software.shtml) (Zhang & Feng, 2004). Data quality control and the calculation process were conducted in strict accordance with the study requirements. Xinjiang is located in the westerly-dominated climatic regime, and the precipitation is impacted by the latitude wave propagation of the mid-latitude atmospheric circulation and the Atlantic Multidecadal Oscillation (AMO) (Huang et al., 2015; Chen et al., 2019), and the drought variability was closely related to AMO and Multivariate ENSO Index (ENSO3.4) events (Yao et al., 2019), Some studies have shown that changes in AMO can also influence climate change in northwest China (Wu, Zhou & Li, 2016; Dong & Dai, 2015; Meehl et al., 2013). The drought and flood events in Xinjiang had significant correlation with the annual North Atlantic Oscillation (NAO), for periods of less than 10 years (Ling et al., 2017). Guan & Yamagata (2003) have demonstrated that the Indian Ocean Dipole (IOD) is at least one possible cause of the abnormal East Asian summer climate, and influenced the precipitation over China. Correlations between various indices (the annual NAO, AMO, ENSO3.4 and IOD) and extreme precipitation events were analyzed. The IOD and NAO values between 1961 and 2018 were collected from the National Climate Center of China (https://cmdp.ncc-cma.net/cn/index.htm), the ENSO3.4 values between 1961 and 2018 were obtained from the monthly index of the National Weather Service Climate Prediction Center of USA (https://origin.cpc.ncep.noaa.gov/products/analysis_monitoring/ensostuff). The AMO values between 1961 and 2018 were obtained from the National Center for Atmospheric Research (https://climatedataguide.ucar.edu/climate-data). The information used in this study was collected from the National Geographic Information Bureau of Surveying and Mapping Standard Map Website (http://bzdt.ch.mnr.gov.cn). Approval was received to download GS (2019) 3333 standard maps, which were reproduced without modification.

In our study, we used extreme precipitation indices to detect and analyze changes in precipitation. There are many definitions of extreme precipitation, and Nicholls & Murray (1999) presented a series of extreme precipitation indices. For the analysis of extreme precipitation indices, it is recommended to use the methods followed by the World Climate Research Programme project operated by the US Climate Variability and Predictability expert team on climate change detection (Easterling et al., 2003). These indices were initially used to study trends in the global climate by Frich et al. (2002), and were subsequently applied in other areas by various researchers (Jiang et al., 2011, 2013; Weili et al., 2015). The nine indices describe different aspects of the precipitation process, which were defined and used to analyze extremes and detect precipitation variations in our study (Table 1). All the selected indices can be roughly divided into four categories (Zhang et al., 2011b; Sillmann et al., 2013).

**Table 1:** Definitions of the precipitation indices.

ID Indicator name definitions units			
Absolute indices			
RX1day	Max 1-day precipitation amount	Monthly maximum 1-day precipitation	mm
RX5day	Max 5-day precipitation amount	Monthly maximum consecutive 5-day precipitation	mm
SDII	Simple daily intensity index	Annual total precipitation divided by the number of wet days (defined as PRCP >= 1.0 mm) in the year	mm/day
PRCPTOT	Annual total wet-day precipitation	Annual total PRCP in wet days (RR >= 1 mm)	mm
Threshold indices			
R10	Number of heavy Precipitation days	Annual count of days when PRCP >= 10 mm	day
Duration indices			
CDD	Consecutive dry days	Maximum number of consecutive days with RR < 1 mm	Day
CWD	Consecutive wet days	Maximum number of consecutive days with RR >= 1 mm	day
Percentile-based threshold indices			
R95p	Very wet days	Annual total PRCP when RR > 95th percentile (from 1961 to 2018)	mm
R99p	Extremely wet days	Annual total PRCP when RR > 99th percentile (from 1961 to 2018)	mm

## Methodology

The arithmetic mean values of each index and precipitation records from the 89 stations from 1961 to 2018 in Xinjiang were used to investigate the impacts of climate extremes. The temporal variation of extreme climate events was determined from a regional annual anomaly series and a linear regression analysis. Since the Mann–Kendall test (M–K test) (Kendall, 1975) is not affected by interference from outliers and it is not necessary to follow a certain sample distribution, this test has often been used to analyze trends in meteorological time series (Gocic & Trajkovic, 2013; Pingale et al., 2014; Deng et al., 2014; Zhang et al., 2019). The M–K mutation test can also be used to detect mutation points in time series data. In our study, the M–K and M–K mutation tests were used to detect changes in precipitation extremes. The spatial distribution maps of extreme precipitation indices are drawn using Kriging difference method in ArcGIS 10.0 to analyze spatial differences of them.

Wavelet analysis is a common tool that is widely used to analyze the periodic variation of climate data within a time series. In our study, it was applied to the extreme precipitation indices (very wet days (R95p) and extremely wet days (R99p)) and the inter-annual changes of the climate indices (NAO, AMO, IOD and ENSO3.4) by decomposing the scale space and frequency in the time series. A continuous wavelet transform was used to obtain the relationship between climate indices and precipitation extreme events in terms of time and frequency. The cross wavelet transform was used to investigate the relationship between selected climate indices (NAO, AMO, IOD and ENSO3.4) and precipitation extreme events, then the wavelet transform coherency (WTC) between two CWTs was used to process the statistical coherence and confidence in terms of noise control. The detailed calculation process for this is given in Torrence & Compo (1998) and Grinsted, Moore & Jevrejeva (2004).

## Results

### Annual precipitation and trends

**Figure 2:**
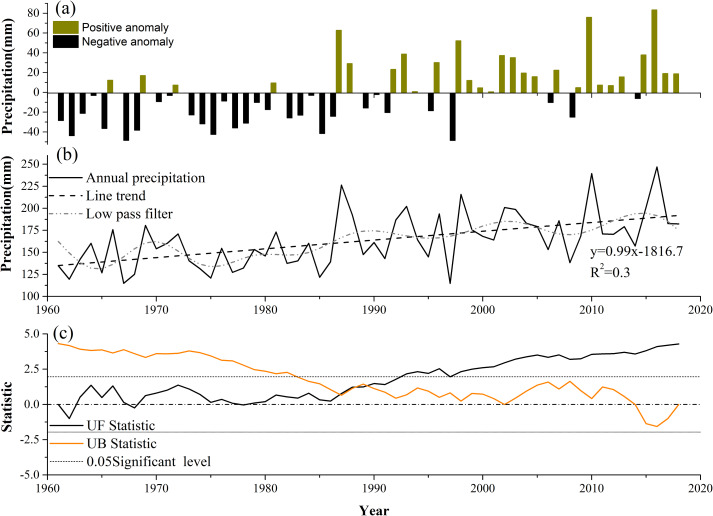
(A) Annual precipitation anomalies in Xinjiang, 1961–2018. (B) Changes of annual precipitation amounts (mm), with the line trend and low pass filter. (C) The results of a Mann–Kendall (M–K) mutation test on the annual precipitation.

The spatial-temporal dynamic characteristics and annual precipitation from 1961 to 2018 in Xinjiang are shown in Fig. 2. The annual precipitation in Xinjiang clearly changed over the 58-year period. The annual precipitation anomalies were based on the average (163 mm) from 1961 to 2018 in Xinjiang. The variance tendency over the 58-year period is shown in Fig. 2A. A number of key results were apparent: (1) The annual precipitation anomaly had surpluses exceeding 20 mm in 13 of these years, while in another 18 years, the annual precipitation anomaly had deficits exceeding 20 mm. Years with negative anomalies were mainly occur in the period from 1961 to 1987, and the positive anomalies mainly occurred after 1988. (2) These 58 years can be divided into two periods: a dry period (1961–1987) and a wet period (after 1988). (3) The driest years in the past 58 years were 1976 and 1997, while 2010 and 2016 were the wettest years. Figure 2B shows the changes in the annual precipitation over time and indicates that precipitation increases at a speed of 0.99 mm per year from 1961–2018.The low-pass filter also indicated that precipitation fluctuation increased, especially after the mid-1980s when the volatility became more intense and frequent. To determine whether the precipitation time series contained a mutation.the results of a Mann–Kendall (M–K) mutation test on the annual precipitation (Fig. 2C). It was found that the precipitation in Xinjiang changed abruptly in 1987, 1988 and 1989, with the results passing a significance test. Combined with the precipitation anomalies in Fig. 2A, it is found that 1987 was a transition year from negative anomalies to positive anomalies, and determined 1987 as the true mutation year.

**Figure 3:**
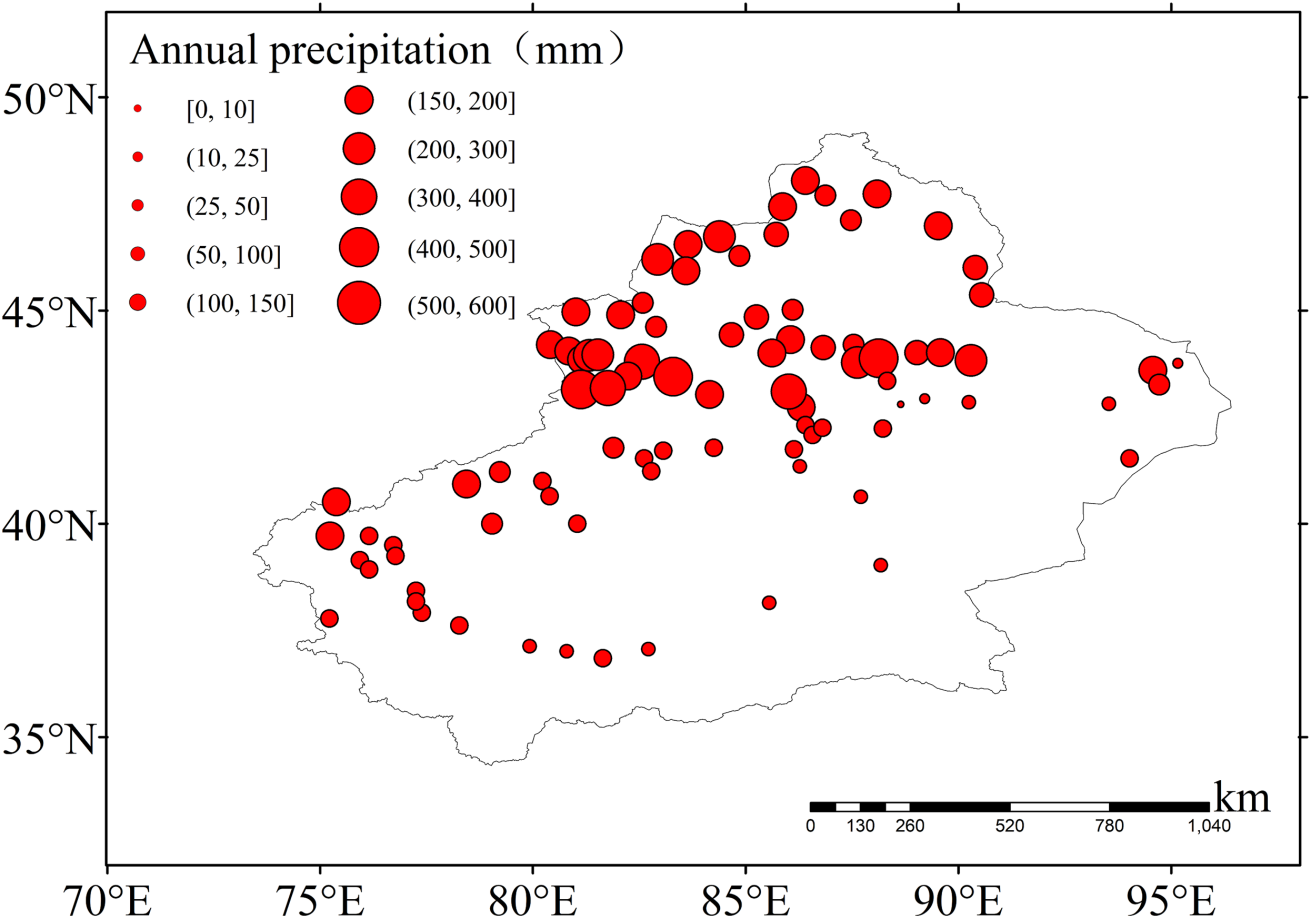
The spatial distribution of average annual precipitation. The size of the red solid circle represents different average annual precipitation.

Figure 3 shows that there was an obvious spatial difference in the annual precipitation of Xinjiang. It was much higher in the mountains compared to the other areas, much higher in the west compared to the east, and much lower in the south compared to the north. The area with the most abundant precipitation was the Tianshan Mountains, with an annual precipitation of more than 600 mm. The precipitation patterns in Xinjiang varied with topography.

### Changes of annual precipitation extremes

#### Abrupt changes in precipitation extremes

**Figure 4:**
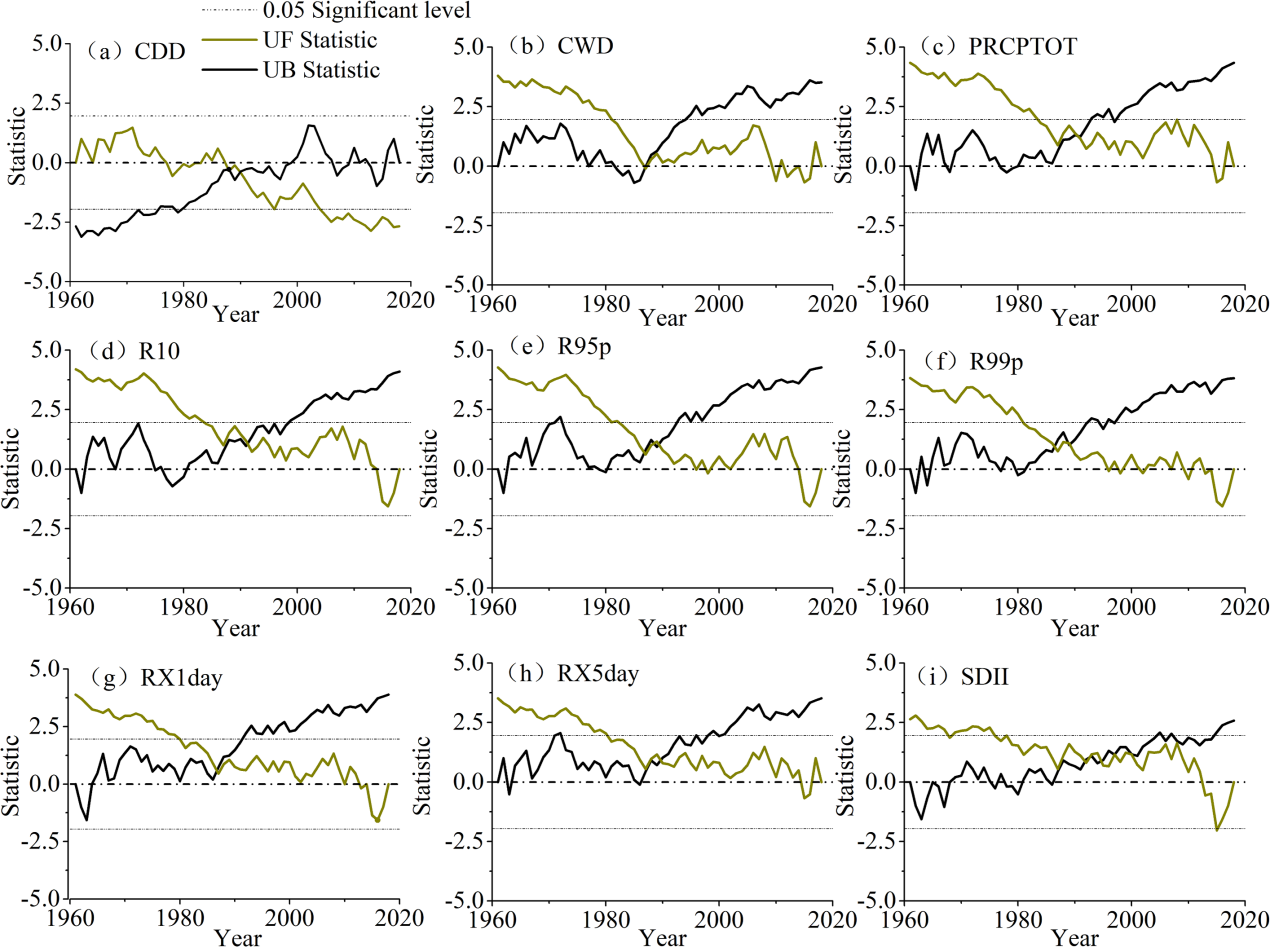
The abrupt changes in precipitation extremes (A, CCD; B, CWD; C, PRCPTOT; D, R10; E, R95p; F, R99p; G, RX1day; H, RX5day; I, SDII).

To detect whether there was a mutation in the precipitation extreme time series from 1961 to 2018, an M–K mutation test was performed on the regional average extreme precipitation indices, as shown in Fig. 4. Nine extreme precipitation indices were selected for use in this study and all of them indicated that there were abrupt changes from 1961 to 2018. However, there were some differences in the timings of the mutation points among the indices. The mutation time, based on the consecutive dry days (CDD) appeared in 1987 and 1989, and the consecutive wet days (CWD) appeared in 1987 (shown in Figs. 4A and 4B). The threshold indices, that is, number of heavy precipitation days (R10) and annual total wet-day precipitation (PRCPTOT) mutated in 1990 (shown in Figs. 4D and 4C). The percentile-based threshold indices, that is, R95p and R99p, changed abruptly in 1990 (R95p appeared in 1989–1990, shown in Figs. 4E and 4F). The mutations based on the other indices, maximum 1-day precipitation amount (RX1day), maximum 5-day precipitation amount (RX5day), and simple daily intensity index (SDII), occurred in 1987, 1990 and 1992, respectively (shown in Figs. 4G, 4H and 4I). The mutations of these indices passed a significance test. Although the mutation times of these indices were different, they were concentrated in the period from 1986 to 1992.

### Variation of the trends in precipitation extremes

**Figure 5:**
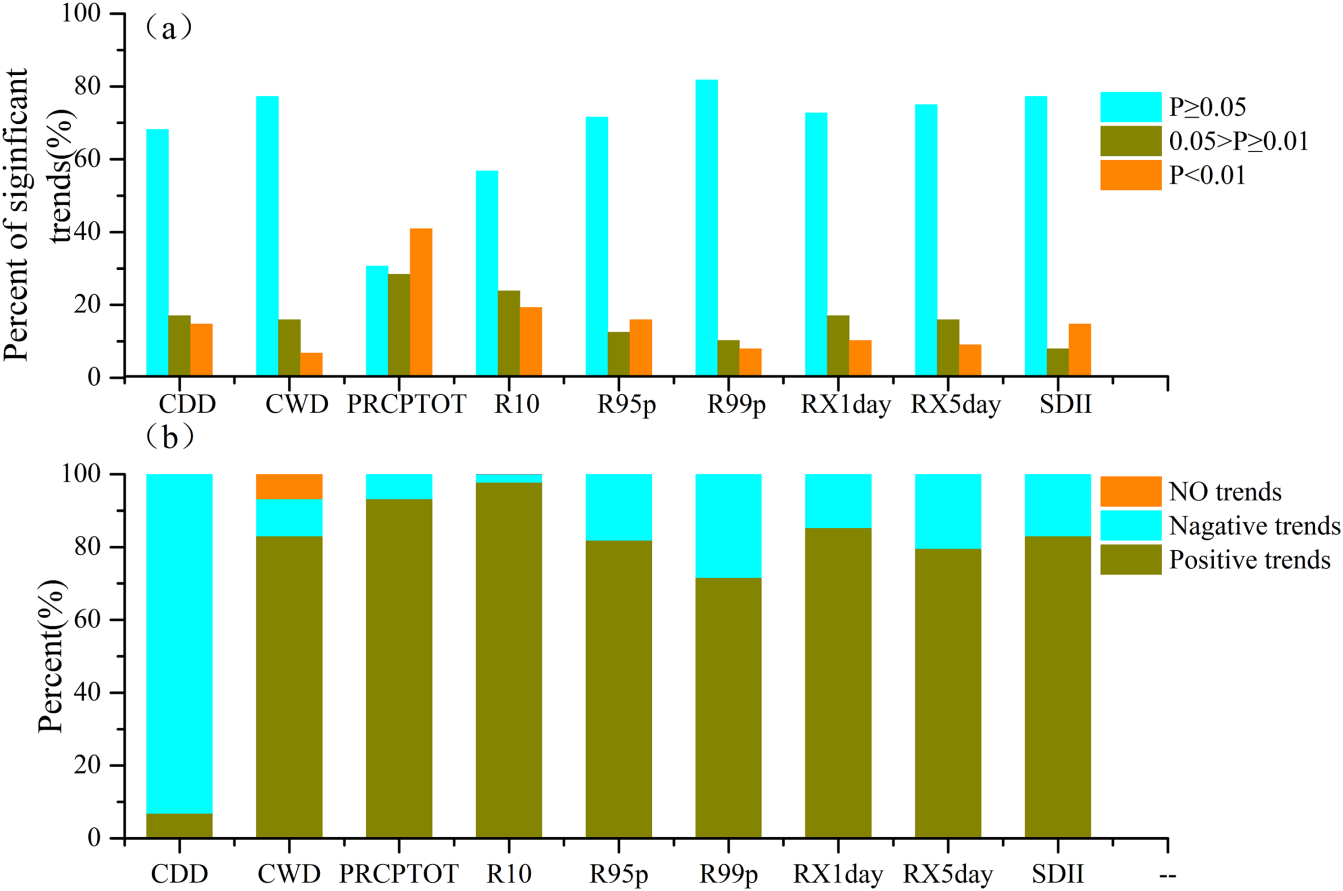
Percentage of all stations with negative and positive trends for each index in Xinjiang from 1961 to 2018. (A) The percentage of results that passed a significance test for each extreme precipitation indices; (B) the percentage of trends for each extreme precipitation indices.

The statistical results of the trends of the extreme precipitation indices for the 89 meteorological stations in Xinjiang from 1961 to 2018 are shown in Fig. 5. Figure 5B shows the percentage of the meteorological stations with zero, positive, and negative trends for each extreme precipitation indices. The percentage of meteorological stations passing the significance level is shown in Fig. 5A. Except for the CDD, which was dominated by a negative trend, all other indices were dominated by positive trends over the 1961–2018 period. The percentage of zero and negative trends was low. It should be noted that the percentage of results that passed a significance test (0.05> *P* ≥ 0.01 and *P* < 0.01) was not high, as shown in Fig. 5A. This indicates that the significant increases of extreme precipitation in Xinjiang were concentrated in some local areas and did not extend across the entire region.

**Figure 6:**
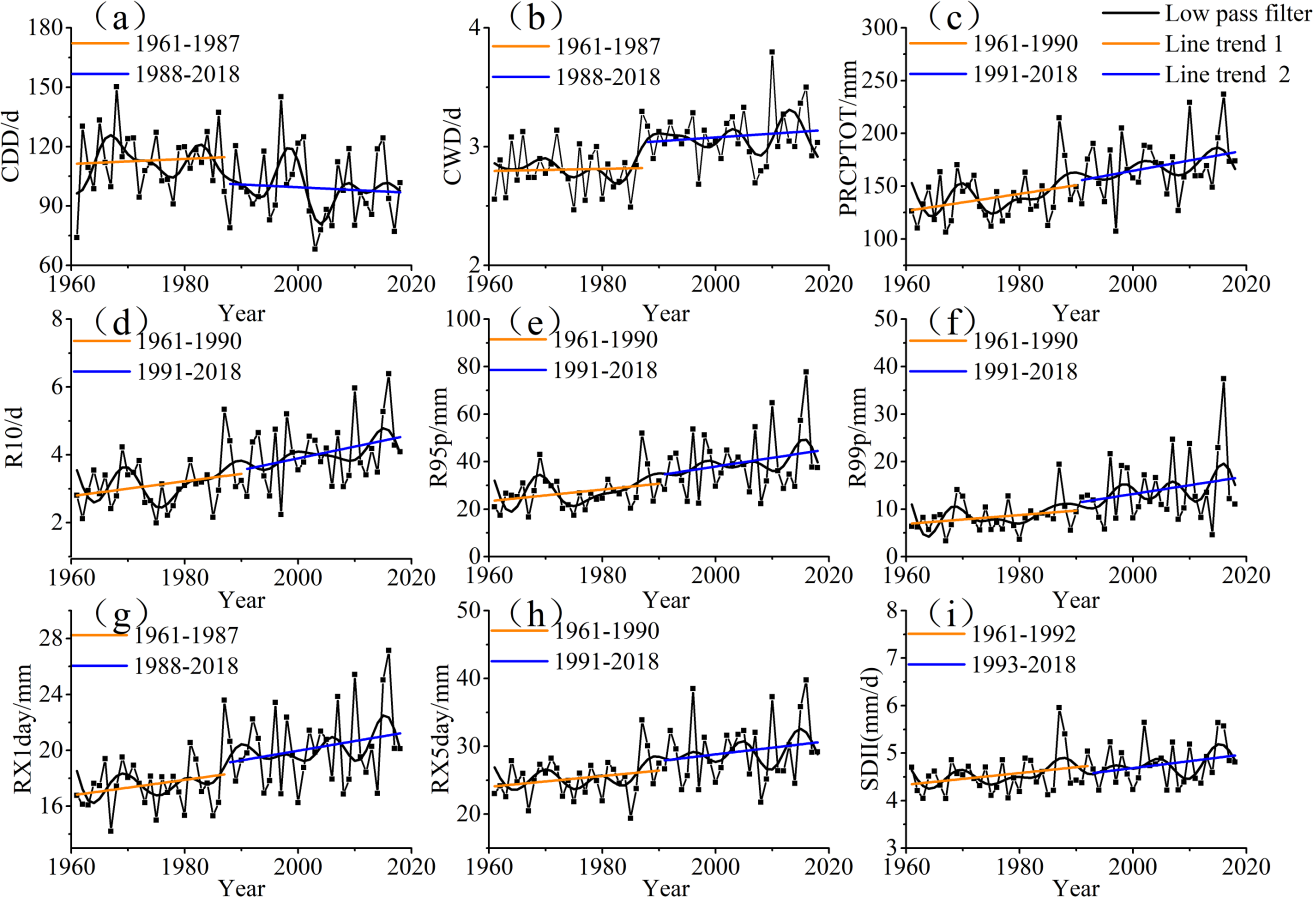
Temporal variation trends of extreme precipitation indices from 1961 to 2018 in Xinjiang (A, CCD; B, CWD; C, PRCPTOT; D, R10; E, R95p; F, R99p; G, RX1day; H, RX5day; I, SDII).

**Figure 7:**
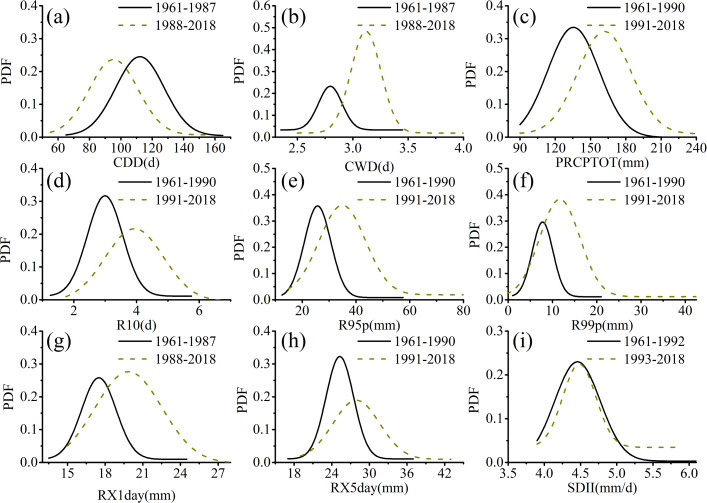
Probability distribution functions of percentile-based annual extreme precipitation events in Xinjiang from 1961 to 2018 (A, CCD; B, CWD; C, PRCPTOT; D, R10; E, R95p; F, R99p; G, RX1day; H, RX5day; I, SDII).

To evaluate the changes in the annual precipitation extreme indices of Xinjiang from 1961 to 2018 based on the mutation test results for each index, a time sequence of each index was constructed (Fig. 6), and the probability distribution functions (PDFs) of the periods before and after the abrupt change in each annual precipitation index were evaluated. The PDFs of the annual precipitation extremes across the two periods are shown in Fig. 7. The CDD and CWD had different trends from 1961 to 2018 (Fig. 6A and 6B). Over the 1961–2018 period, the average CDD was 105.5 days, with a decreasing trend of −3.6 days/10a, but approximately 31.5% (28) of the stations passed a significance test. The slope of the trend was different before and after the mutation (Fig. 6A), with values of −1.26 days/10a for the period from 1961 to 1987 and −1.44 days/10a for the period from 1988 to 2018. The average CWD was 2.96 days and it presented a gradually increasing trend of 0.08 days/10a, but only 22.7% (20) of the stations passed a significance test. The slope of the trends was also noticeably different before and after the mutation, with values of 0.009 days/10a (1961–1987) and 0.03 days/10a (1988–2018). The aforementioned decreasing trend in CDD and increasing trend in CWD could also be determined from the variation in their PDFs (as shown in Figs. 7A and 7B). The PDF curves of CDD were negative from 1988 to 2018, but the CWD moved in the opposite direction and shifted positively. The shifts of the PDF curves clearly indicated a decrease in CDD and increase in CWD. The CDD values corresponding to the maximum probability and maximum values were 113 and 165 days from 1961 to 1987. These values decreased to 95 and 150 days, respectively, during 1988–2018 (Fig. 7A). The CWD values corresponding to the maximum probability and maximum values were approximately 2.7 and 3.3 days from 1961 to 1987, but they increased to 3.2 and 4.2 days, respectively, over 1991–2018 (Fig. 7D). The CDD and CWD trends show that the climate in Xinjiang is becoming wetter.

The RX1day and RX5day values increased over time (Figs. 6G and 6H). For the period from 1961 to 2018, the average RX1day was 18.96 mm, with an increasing trend of 0.84 mm/10a, but only approximately 27% (24) of the stations passed a significance test. The slope of the trend was different before and after the mutation (Fig. 6G), with values of 0.56 mm/10a for the period from 1961 to 1987 and 0.9 mm/10a from 1988 to 2018. In contrast, the average RX5day value was 27.2 mm and it presented a gradually increasing trend of 1.26 mm/10a, but only 25% (22) of the stations had significant growth trends. The slope of the trends, which were also noticeably different before and after the mutation, was 0.8 mm/10a (1961–1990) and 0.98 mm/10a (1991–2018). The increasing trend of the RX1day and RX5day values could also be determined from the variation in their PDFs (as shown in Figs. 7G and 7H). The PDF curves of the RX1day and RX5day values shifted positively. The RX1day values corresponding to the maximum probability and maximum values were approximately 17 and 24.5 mm, respectively, from 1961 to 1987. These figures increased to approximately 19.5 and 28 mm, respectively, during 1988–2018 (Fig. 7G). The maximum probability and maximum values of the RX5day were approximately 25 and 37 mm, respectively, from 1961 to 1987, but they increased to 27 and 43 mm, respectively, over 1991–2018 (Fig. 7H). Similar to the trends of RX1day and RX5day, the PRCPTOT and SDII displayed increasing trends (Figs. 6C and 6I). The average PRCPTOT was 153.5 mm and it displayed an increasing trend of 9.9 mm/10a, with approximately 69% (61) of the stations passing a significance test. The slope of the trend was different before and after the mutation (Fig. 6C), with a value of 8.1 mm/10a for the period from 1961 to 1990 and 0.97 mm/10a from 1991 to 2018, respectively. In contrast, the average SDII was 4.64 mm/d and it presented a gradually increasing trend of 0.09 (mm/d)/10a, but only 22.7% (20) of the stations passed a significance test. The slopes of the trends, which were noticeably different before and after the mutation, were 0.12 (mm/d)/10a for the period from 1961 to 1992 and 0.15 (mm/d)/10a from 1993 to 2018. The PDF curves of PRCPTOT and SDII also moved in a positive direction (Figs. 7C and 7I). The PRCPTOT values corresponding to the maximum probability and maximum values were approximately 135 and 210 mm, respectively, from 1961 to 1990. These values increased to around 165 and 250 mm, respectively, over 1991 to 2018 (Fig. 7C). The SDII value corresponding to the maximum probability and maximum values were approximately 4.5 and 6.1 mm/d, respectively, from 1961 to 1992, but changed to 4.75 and 5.75 mm during 1993–2018. The changes in the four indices indicated that there were increases in extreme precipitation intensity in Xinjiang. The R95p and R99p values were 33.3 and 11.1 mm, respectively, with both indices displaying upward trends at rates of 3.96 and 1.81 mm/10a, in the same order (Figs. 6E and 6F). For the two indices, 28.4% (26) and 18.2% (16) of the stations passed a significance test, with the slope of the trends being 2.45 and 0.93 mm/10a, respectively, for the period from 1961 to 1990 and 3.65 and 1.85 mm/10a, respectively, from 1991 to 2018, that is, before and after the mutation. The R95p values corresponding to the maximum probability and maximum values were approximately 25 and 58 mm, respectively, from 1961 to 1990. These values increased to approximately 40 and 85 mm, respectively, over the latter period (Fig. 7E). Similarly, the maximum probability and maximum values of R99p were around 8 and 20 mm, respectively, from 1961 to 1990. These values increased to approximately 11 and 42 mm, respectively, in the latter period (Fig. 7F). The average R10 value was 3.6 days, and it also exhibited an increasing trend of 0.31 d/10a (Fig. 6D), with 44.3% (39) of the stations displaying statistically significant trends. The R10 values corresponding to the maximum probability and maximum values were around 3 and 5.6 days, respectively, from 1961 to 1990, which increased to approximately 4 and 6.5 days, respectively, over the latter period (Fig. 7D). This indicates that the frequency of extreme precipitation in Xinjiang has increased.

### Spatial variation of extreme precipitation

**Figure 8:**
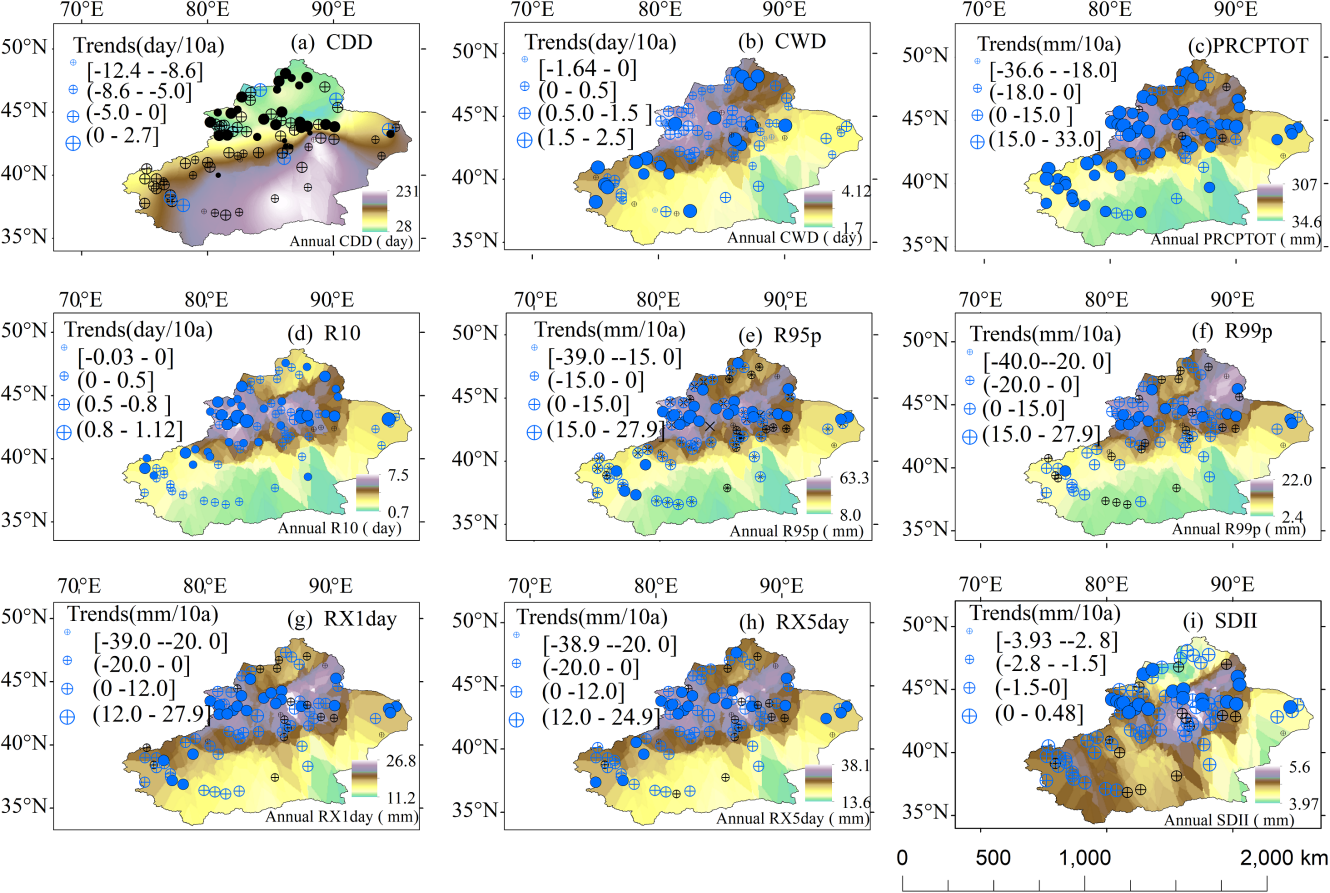
Spatial distribution of the regional averages and trends of the precipitation extreme indices. (A, CCD; B, CWD; C, PRCPTOT; D, R10; E, R95p; F, R99p; G, RX1day; H, RX5day; I, SDII). Black represents a decrease trend and blue represents an increase. Solid means pass the significance test.

The spatial changes in each index value are shown in Fig. 8. It can be seen that the indices indicated a spatial heterogeneity and diversity in extreme precipitation across Xinjiang. Generally, the CDD value was lower in mountainous areas than in other regions. In northern Xinjiang, the CDD value was lower than that of southern Xinjiang, and in the west, it was lower than that of the east. Compared with the CDD, the spatial changes of the other eight indices had the opposite pattern. In a similar manner to the annual precipitation amounts, the value of the other eight indices were greater in mountainous areas than in the other regions. In northern Xinjiang, the values were greater than that in southern Xinjiang, and they were also greater in the west than in the east.

Figure 8 also shows the spatial changes in the trends of the annual series of precipitation indices. There was a significant difference in the spatial distributions of the CDD and CWD trends. The largest areas with a decreasing CDD were in northern Xinjiang and the Tianshan Mountains, with the stations that passed a significance test concentrated in northern Xinjiang. Only six of these stations displayed an increasing trend and they were scattered across the region (Fig. 8A). In contrast to the CDD, for the CWD, the largest area with an increasing trend was western Xinjiang. Only nine of the stations displayed an increasing trend and they were scattered across the western region (Fig. 8B). The spatial distribution of the changing trends of RX1day was very similar to that of RX5day (Figs. 8G and 8H). There were mainly increasing trends for RX1day and RX5day, and the stations that passed a significance test were mainly located in the Tianshan Mountains. However, there were 18 and 19 stations for which a decreasing trend of RX1day and RX5day were observed, respectively, and they were mainly scattered across the eastern Tianshan Mountains and northern Xinjiang. There were certain differences among the spatial distributions of the changing trends of PRCPTOT, R10, and SDII (Figs. 8E, 8D and 8I). Most locations displayed increasing trends and the stations with the largest increases were concentrated in mountainous regions. However, there were clear differences in the spatial distributions of stations that passed a significance test. For PRCPTOT, a large number of stations passed the significance test and they were evenly distributed across the study area. In contrast, for R10 and SDII, only a few stations passed the significance test and they were concentrated in the Tianshan Mountains. However, six stations (PRCPTOT), three stations (R10) and 15 stations (SDII) had a decreasing trend, and they were scattered across the study region. The R95p and R99p mainly increased, and the stations with the largest increase that passed a significance test were mainly located in the Tianshan Mountains (Figs. 8E and 8F). However, there were also 16 stations (R95p) and 25 stations (R99p) in which a decreasing trend was observed. These stations were distributed throughout Xinjiang.

### Period analysis and correlation with climate indices

**Figure 9:**
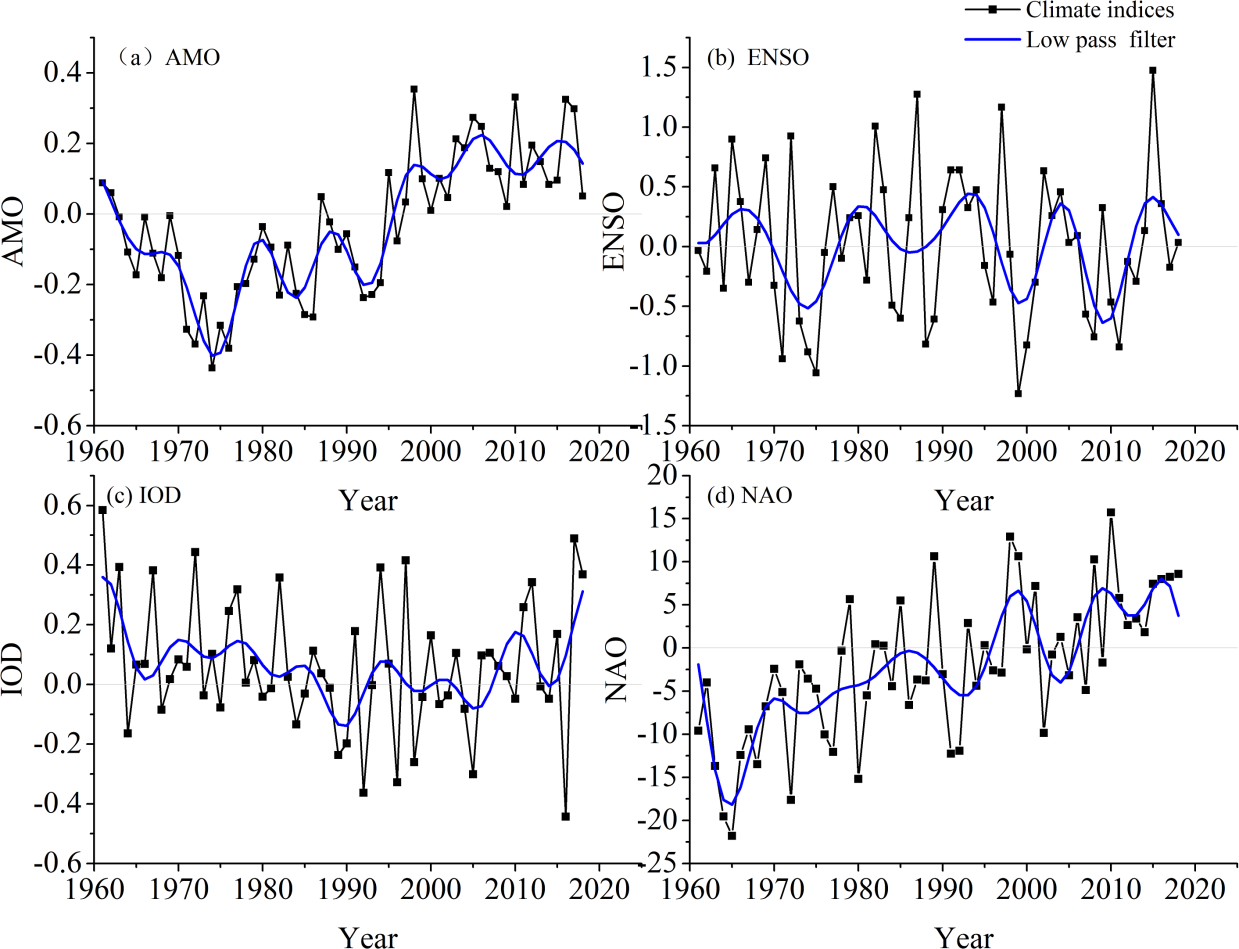
Time series of annual AMO, ENSO, IOD, and NAO from 1961 to 2018 (A, AMO; B, ENSO; C, IOD; D, NAO).

**Figure 10:**
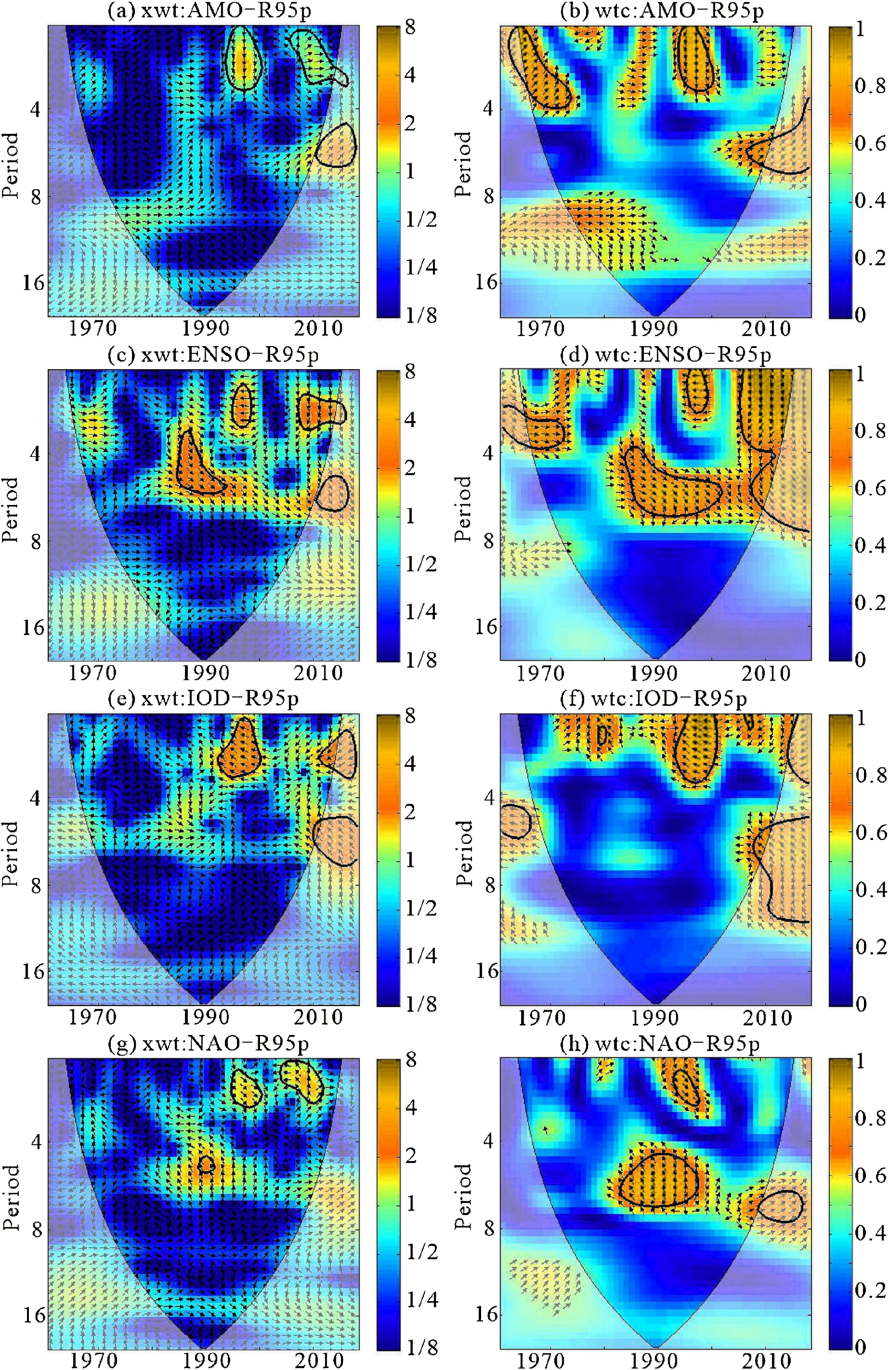
CCross wavelet spectra of the R95p and AMO, R95p and ENSO, R95p and IOD and R95p and NAO time series (A, xwt: AMO-R95p; B, wtc: AMO-R95p; C, xwt: ENSO-R95p; D, wtc: ENSO-R95p; E, xwt: IOD-R95p; F, wtc:IOD-R95p; G, xwt: NAO-R95p; H, wtc: NAO-R95p). ****The thick black contours depict the 5% confidence level of local power relative to orange noise, and the black line is the cone of influence. Right-pointing arrows indicate that the two signals are in phase while left-pointing arrows are for antiphase signals. The coherence power between the climate and precipitation indices is shown as a color gradient of orange to blue, indicating that the correlation between the two series changes from strong to weak. The arrow indicates the difference inphase between the two series. If the arrows are pointing to the right, it indicates that the two time series were in phase, while arrows pointing toward the left mean the time-series are in anti-phase. The up and down arrows indicate that the phases of the two time series differ by 90° (advance or delay, or a 1/4 phase). ****

**Figure 11:**
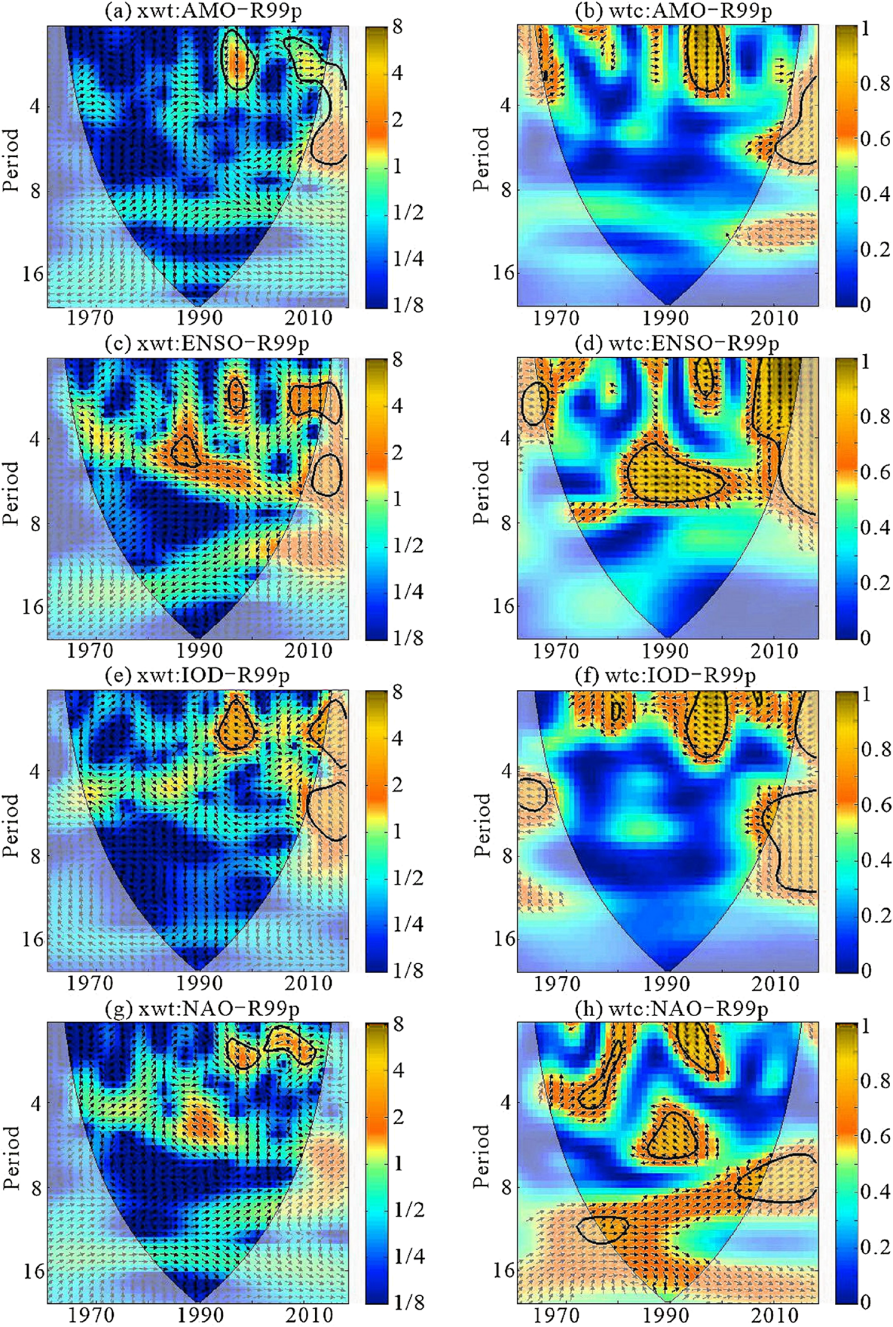
Cross wavelet spectra for the R99p and climate indices (A, xwt:AMO-R99p; B, wtc:AMO-R99p; C, xwt:ENSO-R95p; D, wtc:ENSO-R99p; E, xwt:IOD-R99p; F, wtc:IOD-R99p; G, xwt:NAO-R99p; H, wtc:NAO-R99p).

The time series of annual AMO, ENSO, IOD, and NAO from 1961 to 2018 are shown in Fig. 9, with the four indices showing fluctuations in the different trends. The periodic changes of the extreme precipitation indices (R95p and R99p) and climate indices (AMO, ENSO, IOD, and NAO) in the time series are shown in Figs. 10 and 11. The XWT correlation between R95p and AMO is shown in Fig. 10A. There were two significant power bands: a 1–3-year period from 1992 to 2000 (band (1)) and another from 2005 to 2018 (band (2)), although the direction of the arrows was different. In band (1) the arrows pointed down, while in band (2) the arrows pointed to the right, which indicated that the phases of the two time series differed by 90° and there was a positive correlation between R95p and AMO, respectively. The WTC showed a significant 3–7-year band from 2005 to 2018 and a 1–4-year band around 1962–1975, as shown in Fig. 10B. The arrows pointed down (phases differed by 90°) or to the right (positive phases).

The XWT correlation between R95p and ENSO (Fig. 10C) revealed three significant power bands: a 3–6-year period from 1985 to 1993 (band (1)), a 2–3-year period in the mid-1990s (band (2)), and another from around 2007–2016 (band (3)). The arrows were different in the three bands, with anti-phase conditions in the mid-1990s (band (2)). The WTC between R95p and ENSO showed a 4–7-year period around 1982–2000 (Fig. 10D). It also showed a 2–4-year band from 1961 to 1975. The arrows indicated that the phase of two time series differed by 45° or were in a positive phase. As shown in Fig. 10E, there was one band with a good correlation between IOD and R95p, which revealed a 1–3-year period from 1990 to 1998. The WTC results for the two time series showed that their periodicity and periodic occurrence time were consistent with the XWT results, and the two series were basically in anti-phase (Fig. 10F).

The XWT correlation between R95p and NAO (Fig. 10G) revealed three significant power bands: a 5-year period around 1990, a 2–3-year period in the late 1970s, and a 1–3-year period from 2000 to 2010. There were two significant bands in the WTC between the R95p and NAO (Fig. 10H), which indicated a 5–7-year period from 1982 to 1998 and a 2–3-year period from 1992 to 1998. The direction of the arrows indicated that the time series were neither in phase nor in anti-phase.

Through the analysis of XWT and WTC, the correlation and periodicity of R99p and climate indices were found to be similar to the results of the XWT correlation between the R95p and climate indices (Fig. 11). There were specific correlations between the extreme precipitation events (R95p and R99p) and the four climate indices (AMO, ENSO, IOD and NAO). It was neither a simple linear relationship nor a simple positive or negative relationship, but was more of an advance or delay in phase. This also indicates that the extreme precipitation events in Xinjiang were caused by multiple factors.

## Discussion

Climate change is believed to have influenced changes in hydro-meteorological variables, such as precipitation (Zhang et al., 2005; Scalzitti, Strong & Kochanski, 2016; Xu et al., 2016; Reshmi Mohan et al., 2018). Disasters caused by extreme precipitation have become more frequent and more intense in recent decades (IPCC, 2014). Extreme precipitation has increased in the wet tropics and mid-latitudes (Alexander et al., 2006; IPCC, 2007; Seneviratne et al., 2012; Donat et al., 2013). In mid- to low-latitude countries, such as China. The precipitation is increasing from northwest to southeast China (Zhang et al., 2013), and since 1986 a significant increase in the climate extremes in arid region of northwest China (Deng et al., 2014). Besides, a significant increases in extreme precipitation have been found in western China (Zhai et al., 2005), the precipitation increase was due to the increase in both precipitation frequency and intensity (Ding, Lu & Wang, 2018). the risks of such disasters have also increased as climate change has progressed, studying the spatial distribution of extreme precipitation is not only a scientific exercise, but also has great practical value to the sustainable development of the social economy in China. Many researchers have studied the changing characteristics of precipitation and precipitation extremes (Zhai et al., 1999, 2005; Zhang et al., 2011a, 2013), and it has been observed that precipitation increases in western China are due to increases in the intensity and frequency of precipitation (Zhai et al., 2005). In addition, there is a clear tendency for conditions to become wetter, as reflected by the growing CWD and CCD (Zhang et al., 2013). In this study, we found clear spatial differences in the precipitation extremes of Xinjiang, where the greatest extremes occurred in some mountainous areas, northern Xinjiang and western Xinjiang. Although the mutation times of each of the precipitation extreme indices were different, they were concentrated in the period from 1987 to 1992. The results of this study somewhat differ from earlier studies that reported abrupt changes in 1986 (Chen et al., 2014; Zhang et al., 2019), which were likely caused by differences in the number of stations selected and the study period. The analysis of the indices showed that the climate in Xinjiang was becoming wetter and the frequency and intensity of extreme precipitation events had increased, with these changes being more prominent from the 1990s onward. In terms of the changes in precipitation extremes in our study, our results were similar to those of previous studies (Zhang et al., 2017, 2019; Jiang et al., 2013; Zhang et al., 2012a). Despite the increasing trend of extreme precipitation in most of Xinjiang, a significant differences in its spatial distribution were apparent. These results support the conclusions of previous studies (Wang et al., 2013; Zhang et al., 2019).

Precipitation in northwest China is affected by many factors, including the thermal and dynamic conditions of the Qinghai-Tibet Plateau (Qian, Wu & Liang, 2001a; Qian et al., 2001b), the South Asian high (Wei et al., 2016), the Asian subtropical westerly jet (Zhao et al., 2014b), and the Northwest Pacific subtropical high (Qian et al., 2001b). Some researchers have reported that the changes of latent heat caused by the changes in precipitation in the Indian summer monsoon could affect the precipitation in northwest China (Huang et al., 2014; Chen & Huang, 2012; Ding et al., 2011). The trend toward wetter conditions in northwest China in recent decades can be explained from different perspectives. The increase in precipitation intensity is mainly due to the increase in heavy precipitation events under global warming (Han et al., 2016; Jiang et al., 2013). The increased precipitation in northwest China is also related to changes in the Northern Hemisphere eddy index (Chen & Dai, 2009) and the South Asian monsoon (Zhao et al., 2014a). In addition, the western North Pacific subtropical high and the subtropical high in North America could affect the precipitation in northwest China through transport processes associated with the large-scale water vapor flux (Li et al., 2016). Some studies have shown that changes in PDO and AMO can also influence climate change in northwest China (Wu, Zhou & Li, 2016; Dong & Dai, 2015; Meehl et al., 2013).

In our study, the correlations between the extreme precipitation events (R95p and R99p) and the four climate indices (AMO, ENSO, IOD and NAO) were analyzed. From a statistical perspective, the AMO and NAO show relation to the extreme precipitation in Xinjiang in terms of their correlation and periodicity. This was mainly because Xinjiang is located in the westerly-dominated climatic regime, and precipitation is impacted by the latitude wave propagation of the mid-latitude atmospheric circulation and AMO (Huang et al., 2015; Chen et al., 2019). Extreme precipitation in Xinjiang was also correlated with the periodic changes of ENSO and IOD, indicating that although Xinjiang is located inland, climate change in the region is still affected by the Pacific and Indian oceans, which was also reported by Huang et al. (2015) and Yao et al. (2019). The correlations between the extreme precipitation events (R95p and R99p) and the four climate indices (AMO, ENSO, IOD and NAO) were neither simple linear relationships nor simple positive or negative relationships, but were rather related to an advance or delay in the cycle. This also indicates the complexity of the factors that affect extreme precipitation in Xinjiang, though the exact mechanisms involved in this require further study.

The periodic relationship between extreme precipitation and four climate indices can also indicate the source of water vapor in Xinjiang. Many scholars have studied the water vapor source in Xinjiang, and research pointed out that the moisture sources is imported by a north flow from the Arctic Ocean, a westerly flow from the Atlantic Ocean and along the lakes, and a southwest flow indirectly from the India Ocean and the Bay of Bengal region (Fengqing et al., 2005; Chen et al., 2011; Huang et al., 2013, 2017; Zhao et al., 2014b). Yao, Li & Yang (2018) studied clearly show that water vapor originating from the south, west, and north branches, and water vapor come from the Atlantic Oceans and Central Asia regions has a greater contributed to precipitation in Xinjiang. In arid central Asia, the AMO is associated with the precipitation on a decadal time scale (Huang et al., 2015). The warm and humid water vapor from the tropical Indian Ocean transports into central Asian and converges with the cold air near Balkhash Lake, and this circulation structure is beneficial to precipitation across North Xinjiang. Meanwhile, two types of ENiño have a lag impacts on the summer precipitation over Northwestern Xinjiang (Lu et al., 2019). The warming of the sea surface in the Arabian Sea is conducive to enhanced the southerly flow over the western Indian Ocean which transports warm and humid air from the Indian Ocean into the lowlands between the Iranian Plateau and the Tibetan Plateau. At the meantime, there is a cyclonic circulation model over the Central Asia which brings these wet air masses northward into the Central Asia region, and impact on precipitation events (Zhao & Zhang, 2015).

Xinjiang is a typical arid climate region. The increase of extreme precipitation is beneficial to alleviate drought and reduce the loss caused by drought disaster. However, the harmful aspects of these trends should also be considered. Extreme precipitation events may lead to flood disasters (Kunkel, Andsager & Easterling, 1999). In addition, due to the special geological structure formed by the arid climate in Xinjiang, even small-scale floods may cause landslides.

## Conclusions

Based on 89 weather stations, the nine indices of extreme precipitation were used to analyze the spatiotemporal changes of climate extremes in Xinjiang from 1961 to 2018. In addition, extreme precipitation periodicity and its correlation with climate indices were also examined. The results can be summarized as follows:

1. The extreme precipitation indices indicated some differences in the occurrence time of mutation points, but they were concentrated in the period from 1986 to 1992. The climate in Xinjiang is becoming wetter, and the intensity and frequency of extreme precipitation are increasing, with this trend being more obvious from the 1990s onward.
  Under the background of global warming, the rising frequency of extreme precipitation may be strengthen further in Xinjiang.
2. The extreme precipitation trends showed spatial heterogeneity in Xinjiang. Extreme precipitation was mainly concentrated in mountainous areas, northern Xinjiang, and western Xinjiang. Significant increasing trends of extreme precipitation were also concentrated in the Tianshan mountainous and northern Xinjiang, with these trends being significantly affected by terrain, The extreme precipitation events and their secondary disasters in these areas need further attention.
3. From a statistical perspective, the climate indices (AMO, NAO, ENSO and IOD) were related to extreme precipitation (R95p and R99p) in Xinjiang in terms of correlation and periodicity. However, the correlations between the extreme precipitation indices and climate indices were neither simple linear relationships nor clearly positive or negative relationships; rather, they were related to an advance or delay in the phase. In addition, there were significant periodic changes of approximately 1–3 and 4–7 years in extreme precipitation frequency, which became more obvious from the 1990s onward. There are various sources of water vapor causing extreme precipitation events in Xinjiang, which need further study.

## Supplemental Information

10.7717/peerj.10792/supp-1 Supplemental Information 1 List of 89 stations selected to access dataset at the Meteorological Data Network.

## Additional Information and Declarations

### Data Availability

The following information was supplied regarding data availability: Data were collected from the China Meteorological Data Network: http://data.cma.cn/data/cdcdetail/dataCode/SURF_CLI_CHN_MUL_DAY.html, Data Retrieval was from 1961–2018. Elements selected were daily precipitation and the 89 stations named in a Supplemental File. Due to copyright restrictions, this dataset can be accessed after completing registration at the China Meteorological Data Network.
