# Supplementary material for: Changes in precipitation amounts and extremes across Xinjiang (northwest China) and their connection to climate indices
Source: PeerJ. 2021 Jan 25;9:e10792. doi: 10.7717/peerj.10792 (PMC7842144; doi:10.7717/peerj.10792)
Supplement: Supplemental Information 1 [file peerj-09-10792-s001.docx]

**Table S1**

The selected meteorological stations in the study region

| **Station name** | **WMO number** | **Latitude (°N)** | **Longitude(°E)** | **Altitude(m)** |
| --- | --- | --- | --- | --- |
| Habahe | 51053 | 48.05 | 86.40 | 534 |
| Jimunai | 51059 | 47.43 | 85.87 | 983.9 |
| Buerji | 51060 | 47.70 | 86.87 | 465.5 |
| Fuhai | 51068 | 47.12 | 87.47 | 502 |
| Aletai | 51076 | 47.73 | 88.08 | 736.9 |
| Fuyun | 51087 | 46.98 | 89.52 | 826.6 |
| Tacheng | 51133 | 46.73 | 84.38 | 536.6 |
| Yuming | 51137 | 46.20 | 82.93 | 716.2 |
| Emin | 51145 | 46.55 | 83.65 | 523.3 |
| Hebukesaier | 51156 | 46.78 | 85.72 | 1294.2 |
| Qinghe | 51186 | 46.01 | 90.38 | 1220 |
| Alashankou | 51232 | 45.18 | 82.58 | 286.4 |
| Bole | 51238 | 44.90 | 82.07 | 532.9 |
| Tuoli | 51241 | 45.93 | 83.60 | 1077.7 |
| Kelamayi | 51243 | 46.28 | 84.85 | 445.6 |
| Beitashan | 51288 | 45.37 | 90.53 | 1654.7 |
| Heerguosi | 51328 | 44.20 | 80.42 | 774 |
| Hecheng | 51329 | 44.05 | 80.85 | 641 |
| Wuquan | 51330 | 44.97 | 81.02 | 1353.9 |
| Jinghe | 51334 | 44.62 | 82.90 | 321.2 |
| Wusu | 51346 | 44.43 | 84.67 | 478.3 |
| Paotai | 51352 | 44.85 | 85.25 | 337.8 |
| Mosuowan | 51353 | 45.02 | 86.10 | 347.2 |
| Shihezi | 51356 | 44.32 | 86.05 | 443.7 |
| Shawan | 51357 | 44.00 | 85.62 | 523.2 |
| Caijiahu | 51365 | 44.20 | 87.53 | 441 |
| Hutubi | 51367 | 44.13 | 86.82 | 523.5 |
| Jimusaer | 51378 | 44.02 | 89.02 | 735.4 |
| Qitai | 51379 | 44.02 | 89.57 | 794.2 |
| Cabuchaer | 51430 | 43.85 | 81.15 | 601 |
| Yining | 51431 | 43.95 | 81.33 | 664.3 |
| YIningxian | 51434 | 43.97 | 81.53 | 771 |
| Gongliu | 51435 | 43.47 | 82.23 | 775.6 |
| Wulumuqi | 51463 | 43.78 | 87.62 | 918.7 |
| Dabancheng | 51477 | 43.35 | 88.32 | 1104.2 |
| Mulei | 51482 | 43.83 | 90.28 | 1271 |
| Zhuomaohu | 52112 | 43.77 | 95.13 | 469.8 |
| Kubushi | 51526 | 42.23 | 88.22 | 924 |
| Hejing | 51559 | 42.32 | 86.40 | 1102 |
| Yanqi | 51567 | 42.08 | 86.57 | 1057.2 |
| Heshuo | 51568 | 42.25 | 86.80 | 1086.5 |
| Tuokeshun | 51571 | 42.80 | 88.63 | 2.2 |
| Tulufan | 51573 | 42.93 | 89.20 | 37.2 |
| Shanshan | 51581 | 42.85 | 90.23 | 399.1 |
| Wushen | 51627 | 41.22 | 79.23 | 1396.7 |
| Akesu | 51628 | 41.00 | 80.23 | 1105.3 |
| Paicheng | 51633 | 41.78 | 81.90 | 1230 |
| Xinghe | 51636 | 41.53 | 82.62 | 1014.5 |
| Shaya | 51639 | 41.23 | 82.78 | 981.3 |
| Luntai | 51642 | 41.78 | 84.25 | 977.6 |
| Kuchen | 51644 | 41.72 | 83.07 | 1082.9 |
| Yuli | 51655 | 41.35 | 86.27 | 886.1 |
| Kuerle | 51656 | 41.75 | 86.13 | 932.7 |
| Atushi | 51704 | 39.72 | 76.17 | 1299.3 |
| Jiashi | 51707 | 39.50 | 76.73 | 1213.1 |
| Aketao | 51708 | 39.15 | 75.95 | 1324.9 |
| Yuepuhu | 51717 | 39.25 | 76.78 | 1206.7 |
| Keping | 51720 | 40.01 | 79.05 | 1162.5 |
| Awati | 51722 | 40.65 | 80.40 | 1045.8 |
| Alaer | 51730 | 40.01 | 81.05 | 1013 |
| Tieganlile | 51765 | 40.63 | 87.70 | 847.1 |
| Ruoqiang | 51777 | 39.03 | 88.17 | 889.3 |
| Yingjisha | 51802 | 38.93 | 76.17 | 1298.5 |
| Taishikuergan | 51804 | 37.78 | 75.23 | 3093.7 |
| Shache | 51811 | 38.43 | 77.27 | 1232 |
| Yecheng | 51814 | 37.92 | 77.40 | 1360 |
| Zepu | 51815 | 38.18 | 77.27 | 1275 |
| Pishan | 51818 | 37.62 | 78.28 | 1376.3 |
| Cele | 51826 | 37.02 | 80.80 | 1337 |
| Hetian | 51828 | 37.13 | 79.93 | 1374.7 |
| Minfeng | 51839 | 37.07 | 82.72 | 1409.7 |
| Qiemo | 51855 | 38.15 | 85.55 | 1248.4 |
| Yutian | 51931 | 36.85 | 81.65 | 1423.3 |
| Hami | 52203 | 42.82 | 93.52 | 737.9 |
| Hongliuhe | 52313 | 41.53 | 94.01 | 1701 |
| Nileke | 51433 | 43.80 | 82.57 | 1106.1 |
| Xinyan | 51436 | 43.45 | 83.30 | 929.1 |
| Zhaosu | 51437 | 43.15 | 81.13 | 1854.6 |
| Tekesi | 51438 | 43.18 | 81.77 | 1210.9 |
| Xiaoquzi | 51465 | 43.57 | 87.10 | 2161 |
| Baluntai | 51467 | 42.73 | 86.30 | 1738.3 |
| Tianshandaxigou | 51468 | 43.10 | 86.01 | 3543.8 |
| Tianchi | 51470 | 43.88 | 88.12 | 1935.2 |
| Bayinbuluke | 51542 | 43.03 | 84.15 | 2458.9 |
| Tuernaite | 51701 | 40.52 | 75.40 | 3507.2 |
| Wuqia | 51705 | 39.72 | 75.25 | 2177.5 |
| Aheqi | 51711 | 40.93 | 78.45 | 1986 |
| Balikun | 52101 | 43.60 | 94.55 | 1650.9 |
| Yiwu | 52118 | 43.27 | 94.70 | 1729.5 |
